# Numerical Investigation of the Flow Past a Rotating Golf Ball and Its Comparison with a Rotating Smooth Sphere

**DOI:** 10.1007/s10494-017-9859-1

**Published:** 2017-10-26

**Authors:** Jing Li, Makoto Tsubokura, Masaya Tsunoda

**Affiliations:** 1 Laboratory of Computational Fluid Mechanics, Faculty of Engineering, Hokkaido University, Sapporo, Hokkaido 060-0808 Japan; 2 Computational Fluid Dynamics Laboratory, Department of Computational Science, Graduate School of System Informatics, Kobe University, 1-1 Rokkodai, Nada-ku, Kobe, 657-8501 Japan; 3 Complex Phenomena Unified Simulation Research Team, RIKEN Advanced Institute for Computational Science, 7-1-26 Minatojima-Minami-machi, Chuo-ku, Kobe, Hyogo 650-0047 Japan; 4 Research & Development HQ, Research Dept. 1, Sumitomo Rubber Industries, Ltd. 1-1, 2-chome, Tsutsui-cho, Chuo-ku, Kobe, 651-0071 Japan

**Keywords:** Golf ball, Sphere, Aerodynamics

## Abstract

Large-eddy simulations are conducted for a rotating golf ball and a rotating smooth sphere at a constant rotational speed at the subcritical, critical and supercritical Reynolds numbers. A negative lift force is generated in the critical regime for both models, whereas positive lift forces are generated in the subcritical and supercritical regimes. Detailed analysis on the flow separations on different sides of the models reveals the mechanism of the negative Magnus effect. Further investigation of the unsteady aerodynamics reveals the effect of rotating motion on the development of lateral forces and wake flow structures. It is found that the rotating motion helps to stabilize the resultant lateral forces for both models especially in the supercritical regime.

## Introduction

Golf ball dimples have been discovered to be generally efficient in promoting the drag crisis phenomenon [1–3], i.e. the drag drops at a lower critical Reynolds number for a golf ball when compared to a smooth sphere. On the other hand, a golf ball always spins while flying in real life, hence to investigate the Magnus effect [4] on golf balls is of particular importance.

Davies [5] might be among the first to experimentally investigate the aerodynamic forces of golf balls by dropping the spinning golf balls in a wind tunnel. However, the results provided in his study is limited because the drag and lift forces were not directly measured but calculated based on the drift of the balls, and the negative Magnus effect was not reported for the golf balls although a negative lift force was observed in the smooth sphere cases completed in the same study.

The probably most famous contribution to the aerodynamics of golf balls is the experimental measurement conducted by Bearman and Harvey [1]. The aerodynamic forces exerted on the golf balls were measured over a wide range of Reynolds number and spin parameter using the wind tunnel technique developed in their study, and the results reveal three important phenomena: (1) the golf ball dimples causes the drag force reduction at a lower Reynolds number when compared to a smooth sphere. (2) The reduced drag coefficient barely increases as the Reynolds number further increases in the supercritical regime. It is suggested by Bearman and Harvey that this is because the dimples are effective in tripping boundary layers without causing the thickening of the boundary layer. (3) Under the condition of some specific Reynolds numbers and spin parameters, the negative Magnus force appears on the spinning golf ball. Moreover, Bearman and Harvey estimated the aerodynamic performance of golf balls with different dimple patterns. The result shows that a hexagonally-dimpled ball has a higher lift force and a lower drag force when compared to a spherically-dimpled ball.

Based on the measurements completed by Bearman and Harvey, Choi et al. [2] further investigated the detailed process of how golf ball dimples help to reduce the drag force at a lower Reynolds number when compared to smooth spheres. Using a hot-wire anemometer, they directly measured the streamwise velocity and its fluctuation inside individual dimples located at various angular positions. Based on the measurements, Choi et al. suggested the existence of the local separation bubbles when the flow traverses individual dimples. It is indicated that an unstable shear layer is induced inside the separation bubble, which helps to energize the boundary layer flow and consequently delay the full flow detachment. Moreover, Choi et al. examined the reason that the reduced drag coefficient barely increases as the Reynolds number further increases in the supercritical regime. It is indicated that, with increasing Reynolds number, the shear layer instability inside the local separation bubble appears at further upstream angular positions, whereas the complete flow separation is nearly fixed at the same angular position.

Due to the challenges of computing flow around golf balls [6], the flow behaviors revealed by Choi had not been numerically demonstrated until recently. Smith et al. [3] investigated the flow over a stationary golf ball at both the subcritical and supercritical Reynolds numbers by conducting direct numerical simulations (DNS) within the framework of an immersed boundary approach. The drag reduction of the golf ball was successfully captured in their numerical work, along with a satisfactory agreement of the drag coefficients with the experimental measurements. Moreover, the local separation bubble inside individual dimples proposed by Choi et al. [2] was numerically demonstrated, and the corresponding small-scale turbulent flow structures were visualized.

Beratlis et al. [7] extended the work of Smith et al. [3] to the investigation of flow past rotating golf balls. They conducted the direct numerical simulations of the rotating golf ball with the same spin parameter at four different Reynolds numbers spanning from the subcritical regime to the supercritical regime. The boundary conditions on the golf ball surface were solved based on the Eulerian grid using an embedded boundary formulation. The negative Magnus force was obtained in the simulation results for one of the cases in the critical Reynolds number regime. Based on the measurements of the time-averaged tangential velocities on both sides of the golf ball, Beratlis et al. suggested that the flow separation on the side spinning against the main flow was delayed due to its local instability, which directly contributed to the generation of the negative lift force. This conclusion is analogous to the assumption by Bearman and Harvey [1]. However, although the negative Magnus effect was demonstrated by statistics, no details concerning the development of unsteady forces exerted on the spinning golf ball was provided in the work of Beratlis et al. [7].

More recently, Li et al. [8] investigated the aerodynamics of a stationary golf ball by conducting large-eddy simulations (LES) using more than 140 million unstructured elements. The near-wall flow was fully resolved in their study and the local flow behaviors inside the individual dimples suggested by Choi et al. [2] and Smith et al. [3] were properly reproduced by their numerical simulations. Moreover, Li et al. investigated the unsteady aerodynamics of the stationary golf ball and reported the effects of the dimples on the development of lateral forces and wake structures of the golf ball.

The present study aims to extend the study of Li et al. [8] to a rotating golf ball. Large-eddy simulations (LES) are conducted for a rotating golf ball and a rotating smooth sphere at Reynolds numbers ranging from a subcritical value to a supercritical value. The ordinary and negative Magnus effect on the spinning golf ball are investigated, and the unsteady aerodynamics of the spinning golf ball is discussed in detail.

## Simulation Overview

### Numerical methods

The governing equations solved in the present LES were the spatially filtered continuity and Navier-Stokes equations for viscous incompressible flow:

1 $$ \frac{\partial \overline u_{i}}{\partial x_{i}}=0 $$

2 $$ \frac{\partial \overline u_{i}}{\partial t}+\frac{\partial} {\partial x_{j}}\overline u_{i}\overline u_{j}=-\frac{\partial \overline P} {\partial x_{i}}+2\frac{\partial} {\partial x_{j}}\left( \nu +\nu_{SGS} \right)\overline S_{ij} $$

3 $$ \overline P =\overline p /\rho +(\overline {u_{i}u_{i}} -\overline u_{i}\overline u_{i})/3 $$

where *u*_*i*_, *p*, *?* *?* and *?*_*SGS*_ are respectively the *i*-th velocity component, pressure, density, kinematic viscosity of fluid and the subgrid-scale (SGS) eddy viscosity. The strain rate tensor *S*_*ij*_ was defined as:

4 $$ \overline S_{ij}=\frac{1}{2}\left( \frac{\partial \overline u_{j}}{\partial x_{i}}+\frac{\partial \overline u_{i}}{\partial x_{j}}\right) $$

The dynamic Smagorinsky model [9] was used in the present LES considering its advantages to solving problems with boundary layer transitions [8, 10, 11]. The governing equations were discretized using the vertex-centered finite volume method. The second-order central difference scheme was adopted for the spatial derivatives while 5% of the first-order upwind scheme was blended in the convection term for the sake of numerical stability. The first-order Euler implicit scheme was used for the time advancement and the maximum Courant number was controlled to be less than 1.0 [8]. The Simplified Marker and Cell (SMAC) approach was used for the coupling of the pressure and velocity fields.

The arbitrary Lagrangian-Eulerian (ALE) method proposed by Hirt et al. [12] was adopted to impose the rotating motion on the golf ball. This method allows instantaneously moving vertexes and/or deforming cells of numerical grid during a simulation. More details of this part are presented in Section 2.5.

The open CFD source code “FrontFlow/red” developed in our group under the project “Frontier Simulation Software for Industrial Science” was used in the present LES [13].

### Definition of coordinate system, force coefficient, spin parameter and Reynolds number

**Fig. 1:**
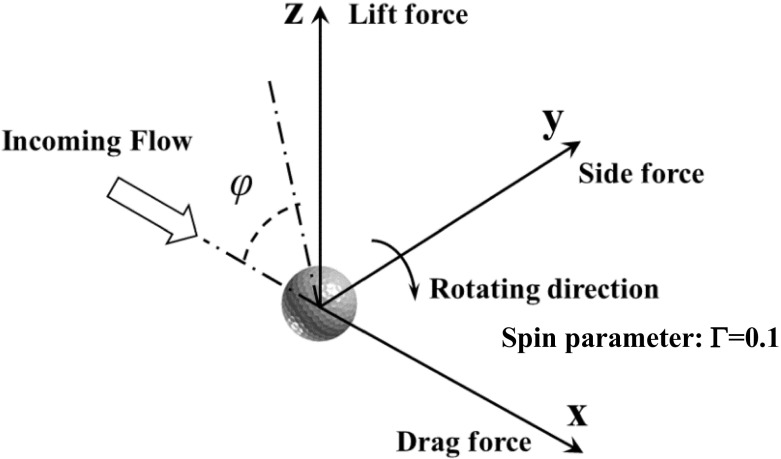
Coordinate system and force directions

Shown in Fig. 1 is the Cartesian coordinate system used in the present study for both the golf ball and smooth sphere. The geometry centers of both models were placed at the position (*x*, *y*, *z*) = (0, 0, 0). The incoming flow approached from the - *x* direction, and the “streamwise direction” was defined as the one from - *x* to + *x* while the drag force direction was defined as along the + *x* direction. For both models, a backspin motion about the “*y*” axis was imposed, and the positive side force direction and positive lift force (Magnus force) direction were respectively defined as along the + *y* and + *z* directions. The angle *f* was defined as the polar angle on the *x* - *z* plane measured from the front stagnation point of the golf ball/smooth sphere. The “azimuthal direction” considered in this study was defined on the *y* - *z* plane.

The drag coefficient *C*_*d*_, side force coefficient *C*_*s*_, lift coefficient *C*_*l*_ and pressure coefficient *C*_*p*_ were respectively defined in the present study as *C*_*d*_ = (2*F*_*d*_)/(*?* *A* *U* ^2^), *C*_*s*_ = (2*F*_*s*_)/(*?* *A* *U* ^2^), *C*_*l*_ = (2*F*_*l*_)/(*?* *A* *U* ^2^) and *C*_*p*_ = *p*/(0.5*?* *U* ^2^), where *F*_*d*_, *F*_*s*_ and *F*_*l*_ respectively represent the drag force, side force and lift force, *A* is the projected frontal area of the golf ball/smooth sphere, *U* is the incoming flow velocity. The spin parameter G which indicates the non-dimensional rotational speed was defined as G = (*?* *D*)/(2*U*), where *?* is the angular velocity and *D* is the diameter of the golf ball/smooth sphere. For all the rotating cases at a constant rotational speed considered in this study, the spin parameter was set as G = 0.1. The value was chosen as the typical flying condition in real golf game. The Reynolds number was calculated in the present study as *R* *e* = *U* *D*/*?*.

### Geometries and mesh generation

**Fig. 2:**
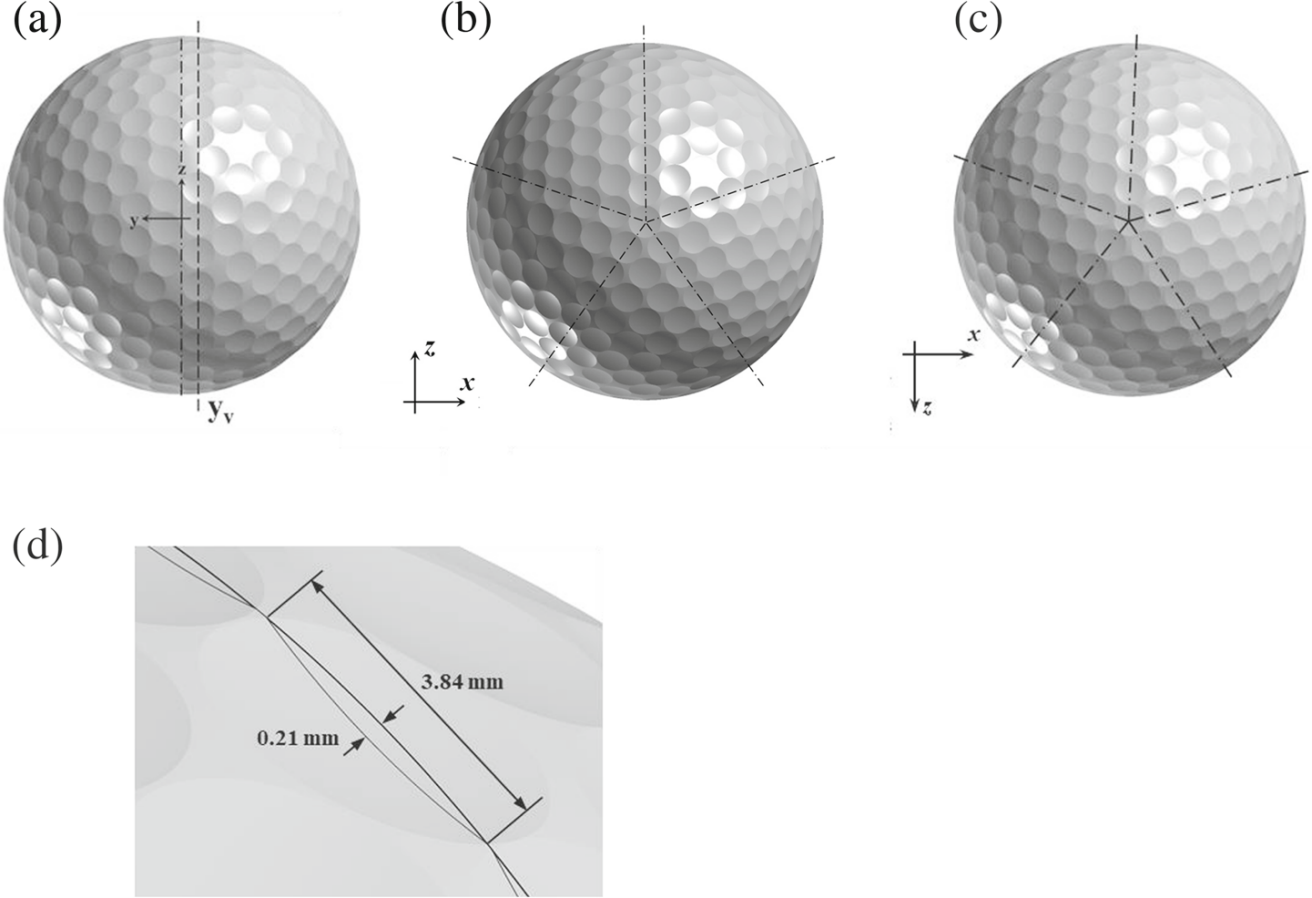
Geometric details of the golf ball visualized on the **a** *y* - *z* view; **b** *x* - *z* view from positive *y*; **c** *x* - *z* view from negative *y*; and **d** dimple geometry

A real golf ball product provided by Sumitomo Rubber Industries [8] and a smooth sphere with the same diameter as the one used by Muto [10] were used in this study. Geometric details of the golf ball are displayed in Fig. 2 through the *y* - *z* view and *x* - *z* view. There are 392 uniform-sized spherical dimples distributed on the surface, and the dimple diameter is approximately 9.0 × 10^-2^ *D*, where *D* is the diameter of the golf ball. The geometry parameter *k*/*D* [1–3, 8] of the present golf ball equals about 0.5 × 10^-2^, where *k* represents the dimple depth. This non-dimensional parameter quantitatively measures the surface roughness of the golf ball. As shown in Fig. 2a, the central joint line of the golf ball (in section *y* = 0) dose not go across any dimples on the surface, therefore an additional visualization plane *y*_*v*_, which intersects the center of the row of dimples closest to the central joint line, was used in some of the interpretations presented in following sections [8].

An unstructured grid was adopted for the mesh generation on both models. Triangular elements were generated on the golf ball/smooth sphere surface and prism layers were allocated along the normal-wall direction starting from the surface mesh. In order to properly capture the boundary layer separation and laminar-turbulent boundary layer transition around the drag crisis region, the numerical grid in the vicinity of the solid wall boundaries must be of sufficient resolution [3, 8, 10]. An estimation of the required grid resolution around a smooth sphere was provided by Muto et al. [10]. They determined the grid resolution based on the laminar boundary layer thickness *d*_*B*_ measured at 90° from the front stagnation point of a sphere, which was estimated as $\delta _{B}=3\times \sqrt {(D\nu )} / {(2U)} $ [14], where *D*, *?* and *U* are respectively the sphere diameter, kinematic viscosity of fluid and incoming flow velocity.

For the present smooth sphere, the mesh size on the sphere surface was equal to *d*_*B*_ while the thickness of the first element of the prism layers was about 1/20 *d*_*B*_. This grid resolution is the same as that applied in Muto’s study [10]. For the present golf ball, an even higher grid resolution was applied considering the requirement of properly reproducing the dimple shape. The mesh size on the golf ball surface was about 2.34 × 10^-3^ *D*, and there were about 40 triangular elements generated across each of the dimples and about 28 prism layers allocated within the distance of dimple depth *k*. Compared with the estimated at *R* *e* = 1.1 × 10^5^, which is the highest Reynolds number considered in the present golf ball case, the surface mesh was smaller than 1/28*δ*_*B*_ while the thickness of the first element of the prism layers was approximately 1/28*δ*_*B*_. This was determined in the past stationary golf ball simulation [8] to maintain the first nearest grid location from the surface less than 1 in wall unit which was defined by the frictional velocity and the kinematic viscosity at *R* *e* = 1.1 × 10^5^.

### Computational domains and boundary conditions

**Fig. 3:**
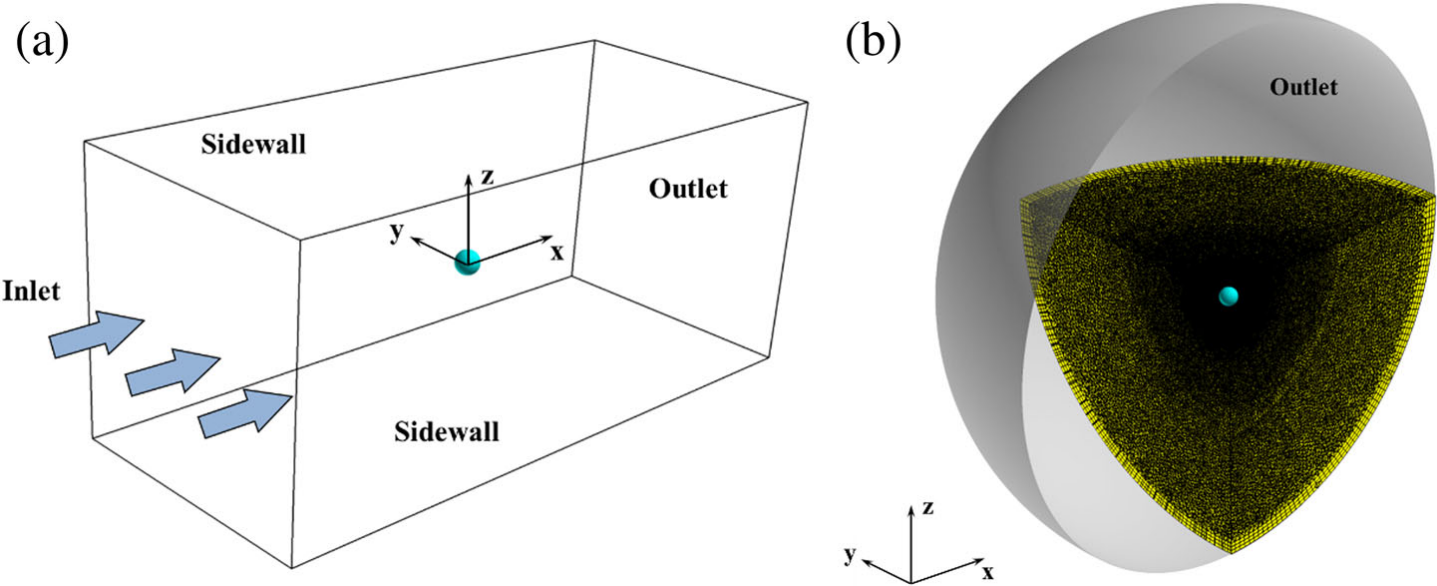
Computational domains used for the **a** rotating smooth sphere; **b** rotating golf ball

Shown in Fig. 3a and b are the computational domains respectively used for the two different rotating models. For the smooth sphere, a rectangular-duct-shaped domain was used and the inertial coordinate system was fixed on the sphere center during the simulation [8]. The scope of this domain is $-13\leqslant x/D\leqslant 13$, $-5.6\leqslant y/D\leqslant 5.6$, and $-5.6\leqslant z/D\leqslant 5.6$, where *D* represents the sphere diameter [8]. For the golf ball, a spherical computational domain was used and the inertial coordinate system was fixed on the “ground” during the simulation. The radius of this domain is *R*_*domain*_ = 15.2*D*, where *D* represents the golf ball diameter. Accordingly, the blockage ratio for the rectangular-duct-shaped domain and the spherical domain are 0.755% and 0.108%, respectively, which are small enough to avoid the effect of flow acceleration around the balls on the aerodynamic forces. The reason that two different computational domains were used was because the rotating motions were imposed on the two models in different ways. More details of this part are presented in the next section. It should be noted that for all the discussions of the golf ball cases presented in this paper, the results are interpreted after being transformed to the coordinate system shown in Figs. 1 and 2.

For the rectangular-duct-shaped domain, a uniform velocity profile of the incoming flow was imposed at the inlet boundary and a zero-gradient condition was imposed at the outlet boundary, whereas a free-slip condition was imposed at all the sidewall boundaries. For the spherical domain, the single external boundary surface was treated as an outlet boundary where the same zero-gradient condition was imposed. On the surfaces of both models, the no-slip condition was imposed [8].

### Ways of imposing rotating motions

The rotating motions were imposed on the smooth sphere and the golf ball in different ways considering their different geometries. For the smooth sphere, which has a homogeneous surface shape, the rotating motion was achieved by directly imposing a rotating speed on the sphere, and the corresponding tangential velocity distribution was computed and imposed on the sphere surface as a boundary condition. This method, however, is not applicable to a rotating golf ball due to the existence of dimples, which results in a continuous change of the surface shape with respect to the approaching flow during rotation.

The above problem of spinning golf balls was solved in the present study by imposing a self-spinning motion on the entire computational domain, including the golf ball surface (solid wall boundary) and the external boundary surface of the domain, using the ALE method. A translational velocity was simultaneously imposed on the entire domain using the same method, which corresponds to the “incoming flow velocity” in the smooth sphere case. At every time step during the simulation, all the numerical cells and vertices moved to a new position (determined by the specific translational and rotational speeds) computed in the inertial coordinate system fixed on the “ground” (see Fig. 3b). This actually simulated the process of “a spinning golf ball flying in the air”. However, this process also resulted in the movement of the numerical cells on the external boundary of the computational domain, thus a domain which has a homogenous external boundary surface was required, such as the spherical domain used in the present study.

## Results

### Statistical features

**Fig. 4:**
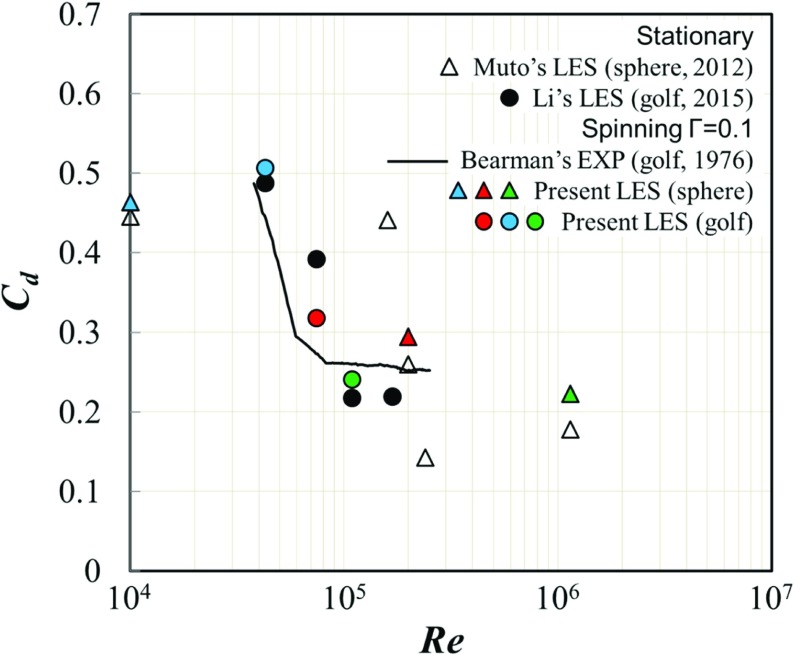
Comparison of the drag coefficient *C*_*d*_ between the rotating golf ball/smooth sphere and the stationary golf ball/smooth sphere; blue symbols: subcritical Reynolds number; red symbols: critical Reynolds number; green symbols: supercritical Reynolds number. Experimental data obtained by Bearman and Harvey [1] at the same spin parameter with different *k*/*D* = 0.9 × 10^-2^ with 250 dimples, while ours are *k*/*D* = 0.5 × 10^-2^ with 392 dimples

The present simulations were conducted respectively for the rotating golf ball at *R* *e* = 4.3 × 10^4^ (subcritical), *R* *e* = 7.5 × 10^4^ (critical), and *R* *e* = 1.1 × 10^5^ (supercritical), and for the rotating smooth sphere at *R* *e* = 1.0 × 10^4^ (subcritical), *R* *e* = 2.0 × 10^5^ (critical), and *R* *e* = 1.14 × 10^6^ (supercritical). Figure 4 compares the drag coefficients of all rotating cases to the corresponding stationary cases [8, 10]. Experimental data obtained by Bearman and Harvey [1] at the same spinning parameter with different dimple geometry (relatively deep depth *k*/*D* = 0.9 × 10^-2^ with 250 dimples compared with our *k*/*D* = 0.5 × 10^-2^ with 392 dimples) are also indicated for reference. It is remarkable that both experimental results and our numerical simulation of the golf ball show drastic decrease of the drag coefficient as the Reynolds number increases, while the drag crisis appears relatively lower Reynolds number region of around 5 × 10^4^ in the experiment compared with our simulation. It is certainly caused by the different dimple geometry and rather deep and large dimple with respect to its ball diameter adopted in the experiment which enhances the transition on the ball surface.

**Fig. 5:**
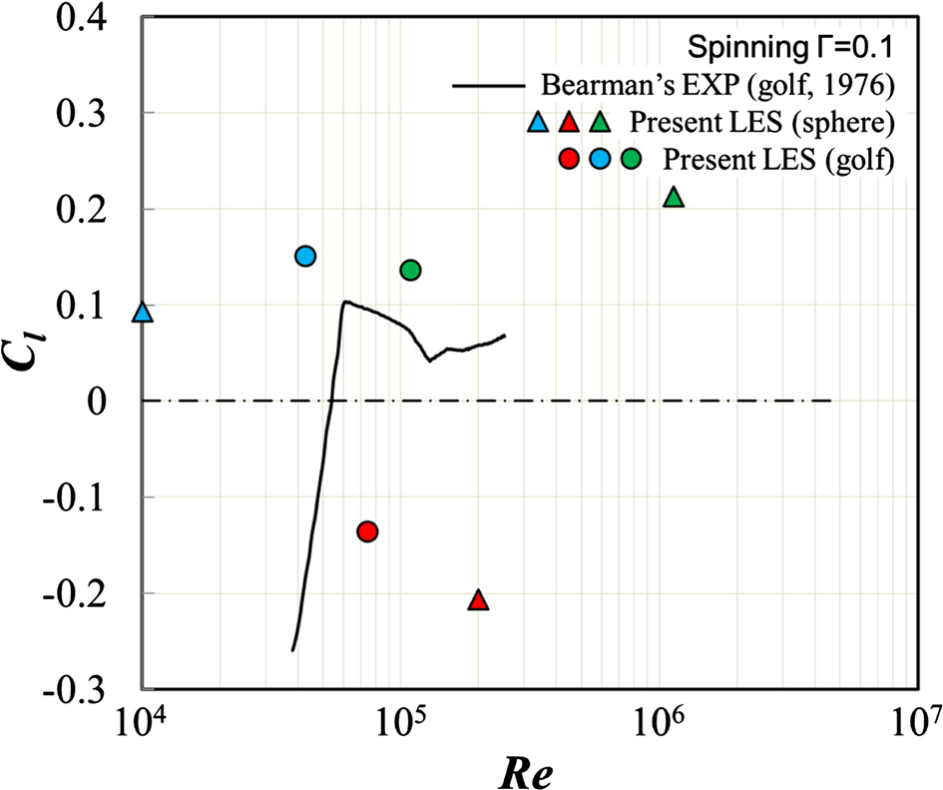
Comparison of the lift coefficient *C*_*l*_ between the rotating golf ball and the rotating smooth sphere; blue symbols: subcritical Reynolds number; red symbols: critical Reynolds number; green symbols: supercritical Reynolds number. Experimental data obtained by Bearman and Harvey [1] at the same spin parameter G = 0.1 with different *k*/*D* = 0.9 × 10^-2^ with 250 dimples, while ours are *k*/*D* = 0.4 × 10^-2^ with 392 dimples

Figure 5 plots the lift coefficients as a function of Reynolds number for all rotating cases, together with Bearman’s experimental data. Table 1 summarizes the time-averaged values and the standard deviations of drag coefficient, side force coefficient and lift coefficient for all rotating cases. The ratios of lift/drag for all rotating cases and the variations of drag coefficient of the rotating models relative to the corresponding stationary models [8] are also provided in this table. With regard to the comparison with past experiment [1], the drastic increase of the lift force from negative to positive as the Reynolds number increases appears relatively lower Reynolds number region around 5 × 10^4^, compared with our numerical result. This is because of the difference of the critical Reynolds number as indicated in Fig. 4. It is remarkable that a negative lift force (the negative Magnus effect) was obtained for both rotating models at the critical Reynolds numbers with current spin parameter (Γ = 0.1), while positive lift forces (the ordinary Magnus effect) were generated for both models at the subcritical and supercritical Reynolds numbers. These results generally agree well with previous literatures [1, 8, 10], but some discrepancies are still shown. First of all, the dependence of the change of the positive lift force on Reynolds number is different for each of the models. For the golf ball, the lift force is slightly smaller at the supercritical Reynolds number when compared to the subcritical Reynolds number, whereas for the smooth sphere, the supercritical lift force is approximately twice as large as the subcritical lift force, as also evidenced in Table 1. On the other hand, in the golf ball case, Beratlis et al. [7] mentioned in their numerical results that “the maximum lift obtained in the beginning of the supercritical regime is higher than that in the subcritical regime”, and the trend is reversed to our results. The possible explanation of this qualitative disagreement comes from the difference of critical Reynolds number between these two cases (around 8 × 10^4^ in our case and 5 × 10^4^ in [7]). This critical Reynolds number difference is likely caused by the difference of dimple geometry between them (our dimple diameter and depth are 9.0 × 10^-2^ *D* and 0.5 × 10^-2^ *D*, respectively, while those of [7] are 1.0 × 10^-1^ *D* and 0.63 × 10^-2^ *D*). Effect of the dimple parameters on the magnitude of the lift coefficient around the critical Reynolds number is remained in the future studies.

**Table 1:** Summary of the time-averaged value (mean) and standard deviation (s.d.) of drag coefficient *C*_*d*_, side force coefficient *C*_*s*_, lift coefficient *C*_*l*_, and the ratio of lift/drag (*C*_*l*_/*C*_*d*_) of the rotating models, and the change of drag coefficient of the rotating models (*C*_*d*_) relative to the corresponding stationary models (*C*_*d*_*sta*_) [8]

		Golf ball			Smooth sphere		
		*R* *e* = 4.3 × 10^4^	*R* *e* = 7.5 × 10^4^	*R* *e* = 1.1 × 10^5^	*R* *e* = 1.0 × 10^4^	*R* *e* = 2.0 × 10^5^	*R* *e* = 1.14 × 10^6^
		(subcritical)	(critical)	(supercritical)	(subcritical)	(critical)	(supercritical)
*C*_*d*_	Mean	0.5059	0.3168	0.2397	0.4635	0.2944	0.2229
	s.d.	0.0215	0.0116	0.0058	0.0107	0.0474	0.0176
*C*_*s*_	Mean	-0.0304	0.0386	-0.0211	0.0040	0.0524	0.0145
	s.d.	0.0243	0.0426	0.0262	0.0186	0.0352	0.0255
*C*_*l*_	Mean	0.1498	-0.1367	0.1353	0.0929	-0.2067	0.2128
	s.d.	0.0392	0.0414	0.0178	0.0266	0.0907	0.0220
*C*_*l*_/*C*_*d*_		0.2961	-0.4315	0.5646	0.2005	-0.7023	0.9549
(*C*_*d*_ - *C*_*d*_*sta*_)		3.8962	-18.9972	10.5440	4.1526	13.2262	25.2017
/*C*_*d*_*sta*_ [%]							

The shifting of the drag coefficient as a result of the rotating motion is depicted in Fig. 4 for both models. For the rotating smooth sphere, the drag coefficients slightly increase at all the three different Reynolds numbers when compared to the non-rotating smooth sphere [10]. Similar features are also observed for the drag force shifting of the rotating golf ball at the subcritical and supercritical Reynolds numbers. At the critical Reynolds number, however, the drag coefficient of the rotating golf ball drops when compared to the non-rotating golf ball [8]. The main reason is supposed to be the shift of the separation point in the downstream direction on the backward rotation side against the main flow as the local Reynolds number increases and effect of turbulence enhancement becomes remarkable, which is contrarily to the forward rotation side. However, this result shows disagreement with Bearman’s [1] work. It is most likely that this discrepancy is due to the different golf balls used by us and by Bearman, and probably is also affected by the Reynolds numbers and spin parameters. Thus effect of the dimple geometry and its Reynolds number effect on the separation-point shift should be studied further to understand this drag coefficient drop in the critical region.

Another interesting thing is, although the drag coefficients of both models decrease as the Reynolds numbers increase from the subcritical values to the supercritical values, the magnitude of the lift coefficient decreases for the rotating golf ball, whereas it increases for the rotating smooth sphere. This further results in a difference in the variation of the ratio of *C*_*l*_/*C*_*d*_ between the two models. As listed in Table 1, for both models, the magnitude of *C*_*l*_/*C*_*d*_ generally increases with Reynolds number. However, the increment of the *C*_*l*_/*C*_*d*_ of the golf ball is visibly smaller when compared to the smooth sphere. In the supercritical regime, particularly, the lift coefficient of the golf ball is about 40% smaller than that of the smooth sphere while the drag coefficients of both models stay close to each other. This further indicates a suppression of the lift force acting on the rotating golf ball in the supercritical regime, as also observed for the non-rotating golf ball by Li et al. [8].

### Flow visualization around the rotating golf ball and the rotating smooth sphere

**Fig. 6:**
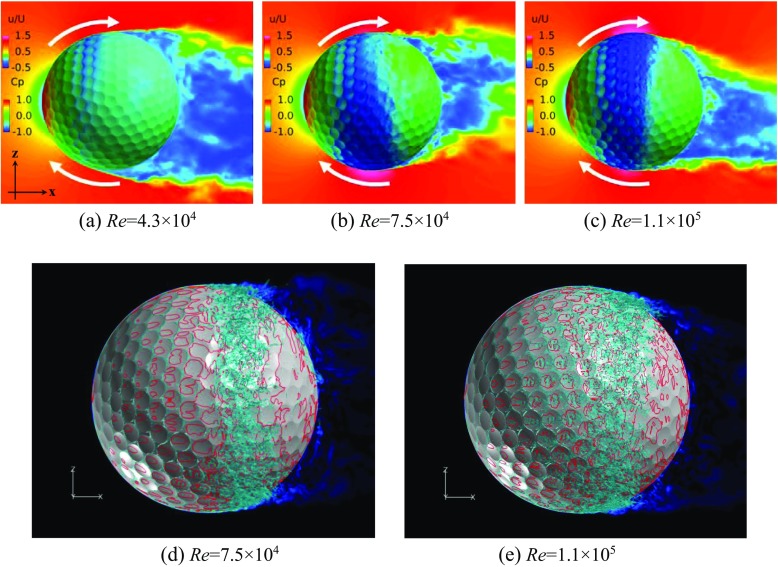
Instantaneous surface pressure distribution and non-dimensional streamwise velocity distribution in the wake area of the rotating golf ball (above), and zero skin friction on the surface (red line) and iso-surface of the Q criterion (below) at the: **a** subcritical, **b**, **d** critical, **c**, **e** supercritical Reynolds numbers; incoming flow was from - *x* to + *x*; the backspin motion was imposed about ‘*y*’ axis; the velocity distribution is viewed in section *y* = 0

**Fig. 7:**
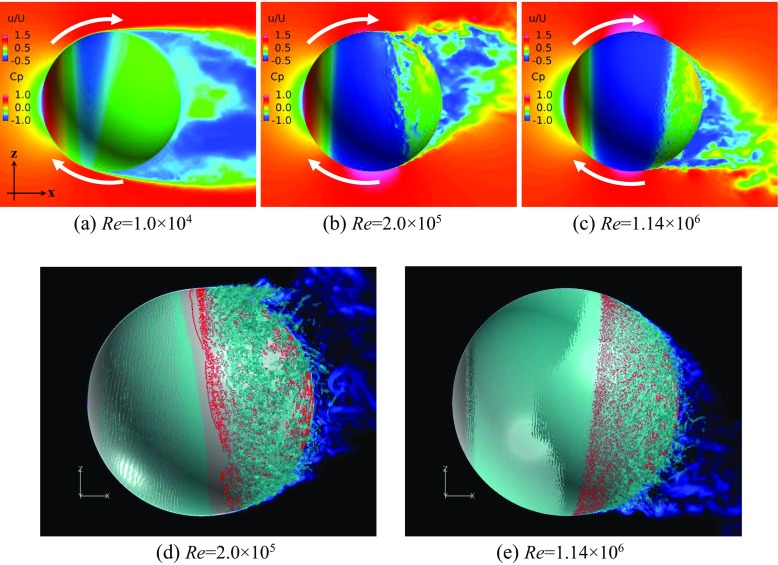
Instantaneous surface pressure distribution and non-dimensional streamwise velocity distribution in the wake area of the rotating smooth sphere (above), and zero skin friction on the surface (red line) and iso-surface of the Q criterion (below) at the: **a** subcritical, **b**, **d** critical, **c**, **e** supercritical Reynolds numbers; incoming flow was from - *x* to + *x*; the backspin motion was imposed about ‘*y*’ axis; the velocity distribution is viewed in section *y* = 0

**Fig. 8:**
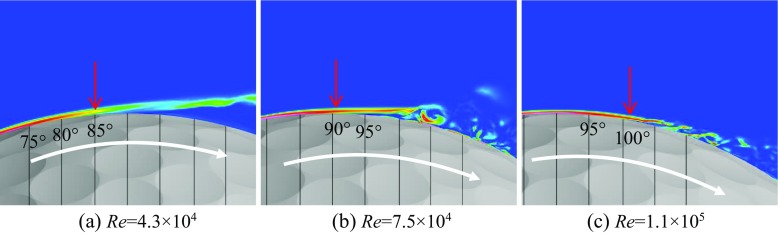
Contours of the instantaneous azimuthal vorticity viewed in section *y* = 0 around the separation point on the top side of the corresponding rotating golf balls shown in Fig. 6; the red arrows indicate the positions of flow separation

**Fig. 9:**
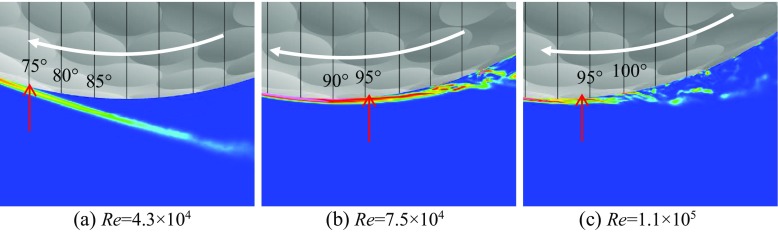
Contours of the instantaneous azimuthal vorticity viewed in section *y* = 0 around the separation point on the bottom side of the corresponding rotating golf balls shown in Fig. 6; the red arrows indicate the positions of flow separation

**Fig. 10:**
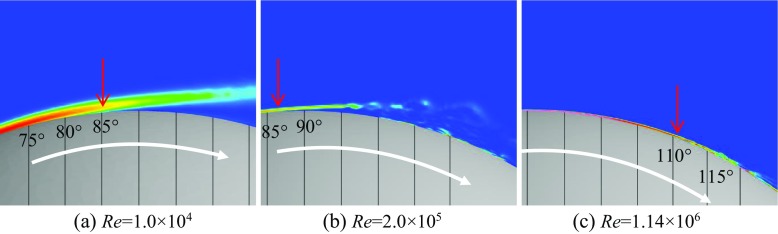
Contours of the instantaneous azimuthal vorticity viewed in section *y* = 0 around the separation point on the top side of the corresponding rotating smooth spheres shown in Fig. 7; the red arrows indicate the positions of flow separation

**Fig. 11:**
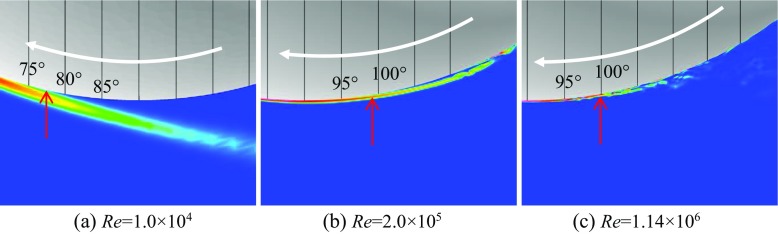
Contours of the instantaneous azimuthal vorticity viewed in section *y* = 0 around the separation point on the bottom side of the corresponding rotating smooth spheres shown in Fig. 7; the red arrows indicate the positions of flow separation

Shown in Figs. 6 and 7 are respectively the instantaneous surface pressure distribution and non-dimensional streamwise velocity distribution in the flow field of the rotating golf ball and the rotating smooth sphere (above), and zero skin friction line on the surface and iso-surface of the Q criterion (below). The zero skin friction line indicates the local separation line on the surface. Figures 8 and 9 display the instantaneous azimuthal vorticity around the flow separation points respectively on the top side and the bottom side of the golf ball. Figures 10 and 11 display the instantaneous azimuthal vorticity around the flow separation points respectively on the top side and the bottom side of the smooth sphere. Due to the backspin motion, the top sides of both models spin in the direction moving with the approaching flow, whereas the bottom sides of both models spin in the direction moving against the approaching flow.

In the subcritical regime, as indicated in the figures, the boundary layers on both the top side and the bottom side of the models remain laminar. One can observe that the shear layers developed after the full flow separation become unstable as they travel further downstream, which is very similar to that observed in the corresponding non-rotating cases [8]. However, the rotating motion leads to an obvious difference in the position of flow separation between the top and the bottom sides. For the rotating golf ball, the flow detaches around 85° on the top side while it detaches around 75° on the bottom side. For the rotating smooth sphere, similarly, the flow separates at a position further downstream on the top side (about 85°) than on the bottom side (about 78°). As a result of these asymmetries, the wake flows behind the rear surfaces of both models are deflected downwards.

In the supercritical regime, the boundary layers on both the top side and the bottom side of the models become turbulent, although for the golf ball it is mainly induced by the perturbation caused by the dimples [1–3, 7, 8], whereas for the smooth sphere it is mainly due to the increase in Reynolds number. As indicated in the figures, the boundary layers exhibit noticeable instabilities before the full flow separation, and shed small-scale vortices. One can also observe that the shear layers become thinner as a result of the high Reynolds numbers. However, the flow separation in the supercritical regime shows a similar pattern as the subcritical regime, despite the fact that the boundary layers are of characteristics totally different from each other. It can be seen that for both models at the supercritical Reynolds numbers, the flow stays attached on the boundary surface for a longer distance on the top side than on the bottom side, which likewise results in a downward-deflected wake flow behind the rear surfaces of the models.

In the critical regime, the boundary layers on the top side and the bottom side exist in different states for both models. On the top side, the boundary layers do not become noticeably unstable or shed vortices that are experiencing Kelvin-Helmholtz instability until they travel some distance downstream from the separation points. These features are very similar to those exhibited in the subcritical regime, although the positions where the shear layers start to become unstable are generally closer to the separation points at the critical Reynolds numbers. On the bottom side, the boundary layer of the smooth sphere first separates after it passes the equator but reattaches to the surface at a position further downstream; for the golf ball, similarly, the near-wall flow shows the features typical of the turbulent boundary layer exhibited in the supercritical case. As a result of the different boundary layer states, the flow passing over both models in the critical regime detaches at a position further downstream on the bottom side than on the top side, which leads to an upward-deflected wake flow behind the rear surfaces of the models.

### Mechanism of the ordinary and negative Magnus effect

**Fig. 12:**
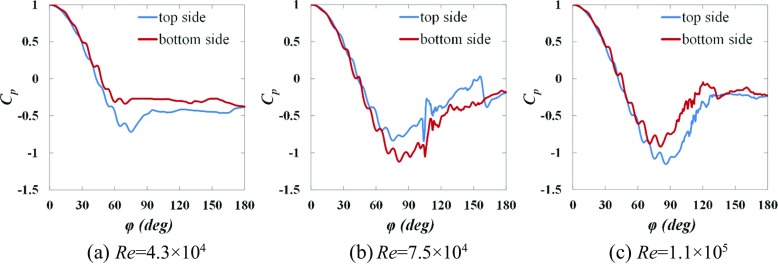
Instantaneous surface pressure distribution obtained in section *y* = 0 respectively along the top side and the bottom side of the golf balls displayed in Fig. 6

**Fig. 13:**
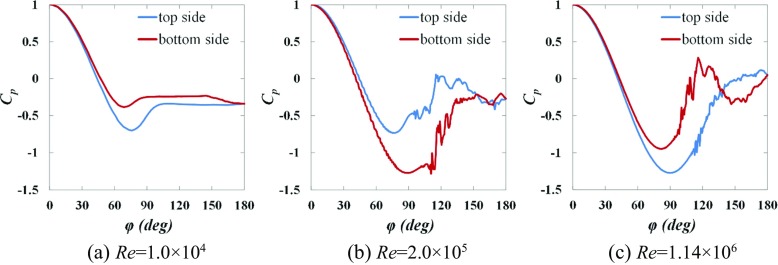
Instantaneous surface pressure distribution obtained in section *y* = 0 respectively along the top side and the bottom side of the smooth spheres displayed in Fig. 7

Figures 12 and 13 respectively show the instantaneous surface pressure coefficients obtained in section *y* = 0 along the top side and the bottom side of the rotating golf ball and the rotating smooth sphere.

As discussed in Section 3.2, for both models in the subcritical and supercritical regimes, the boundary layers on the top side and the bottom side exist in the same state (both sides are laminar or turbulent) but the flow stays attached on the top side for a longer distance. This is attributed to the difference between the effects of the rotating motion on the flow passing over the two different sides. In particular, the near-wall flow gains momentum on the top sides as the geometries rotate with the approaching flow, whereas it loses momentum on the bottom sides as the geometries rotate against the approaching flow. With the higher momentum, the near-wall flow on the top side is able to overcome the adverse pressure gradient more and separate at a position further downstream than on the bottom side. This consequently results in the pressure on the top side experiencing a steeper drop and a lower minimum value than that on the bottom side, as plotted in Figs. 12a, c and 13a, c, which consequently gives rise to a positive lift force acting on the models, i.e. the ordinary Magnus effect.

In contrast, for both models in the critical regime, the boundary layer stays laminar on the top side, whereas it becomes turbulent on the bottom side. This is attributed to the difference in the relative Reynolds number between the two different sides, which is calculated based on the free stream velocity with respect to the spinning surface. In particular, the relative Reynolds number is locally higher on the bottom side than on the top side due to the backspin motion, which makes the flow passing over the bottom side more sensitive to perturbations. As a result, the laminar-turbulent boundary layer transition is promoted on the bottom side, whereas it is accordingly suppressed on the top side due to a lower relative Reynolds number. As a result of the turbulent state, the flow on the bottom side has a higher momentum in the near-wall region and hence separates at a position further downstream than on the top side. This consequently leads to a lower pressure on the bottom side than on the top side in the majority of angular positions, as shown in Figs. 12b and 13b, which consequently produces a negative lift force acting on the models, i.e. the negative Magnus effect.

**Fig. 14:**
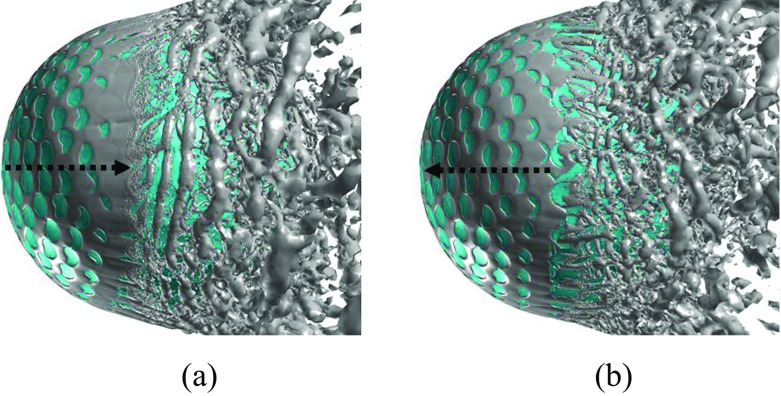
Visualization of the instantaneous vortical structures around the golf ball using ISO surface of Q respectively viewed from the: **a** top view; **b** bottom view; *R* *e* = 4.3 × 10^4^, subcritical

**Fig. 15:**
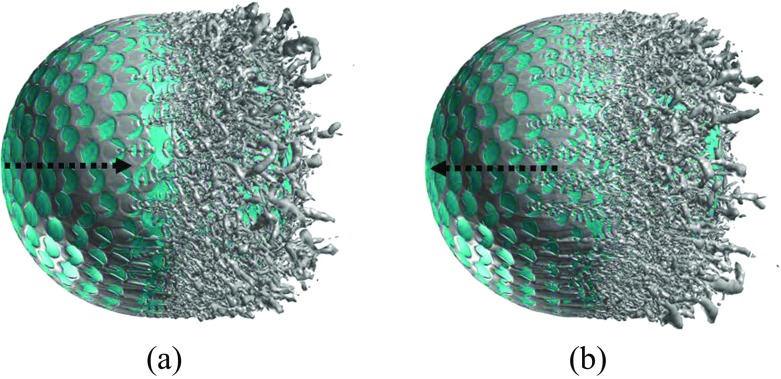
Visualization of the instantaneous vortical structures around the golf ball using ISO surface of Q respectively viewed from the: **a** top view; **b** bottom view; *R* *e* = 7.5 × 10^4^, critical

**Fig. 16:**
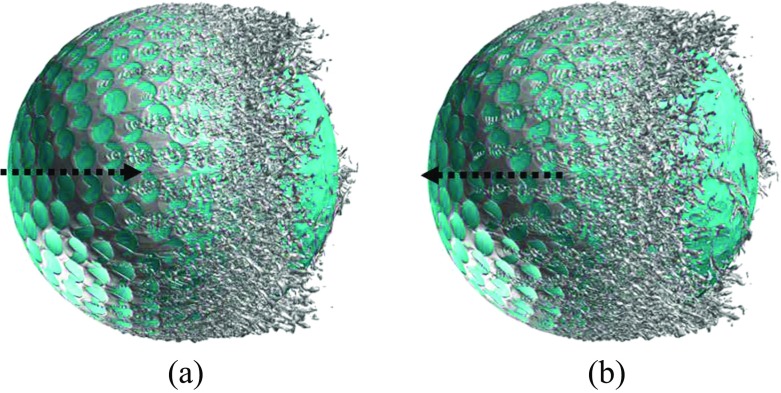
Visualization of the instantaneous vortical structures around the golf ball using ISO surface of Q respectively viewed from the: **a** top view; **b** bottom view; *R* *e* = 1.1 × 10^5^, supercritical

Further insights into the boundary layer separations on the two different sides of the rotating golf ball are provided in Figs. 14, 15 and 16 by visualizing the three-dimensional flow structures in the near wall region respectively from the top view and the bottom view. As indicated in Fig. 14, in the subcritical regime, the flows on both sides of the golf ball barely experience any local oscillations while traversing the dimples before full separation. As indicated in Fig. 16, in the supercritical regime, the flows on both sides of the golf ball experience visible local oscillations and generate small-scale vortices inside the dimples before full detachment. These behaviors are typical of the turbulent boundary layers of a golf ball at supercritical Reynolds numbers [2, 3, 8]. It is also seen from this figure that the higher relative Reynolds number on the bottom side gives rise to the local shear layer instability inside the dimples at positions further upstream than on the top side. As indicated in Fig. 15, in the critical regime, the flow on the top side behaves similarly as the subcritical boundary layer, whereas on the bottom side the local oscillations and small-scale vortices inside the dimples are clearly identified, as exhibited in the supercritical boundary layer. These features further demonstrate the mechanism responsible for the negative Magnus effect.

### Development of the unsteady aerodynamic forces and wake flow structures

#### Transient lateral forces and wake flow structures in the subcritical regime

**Fig. 17:**
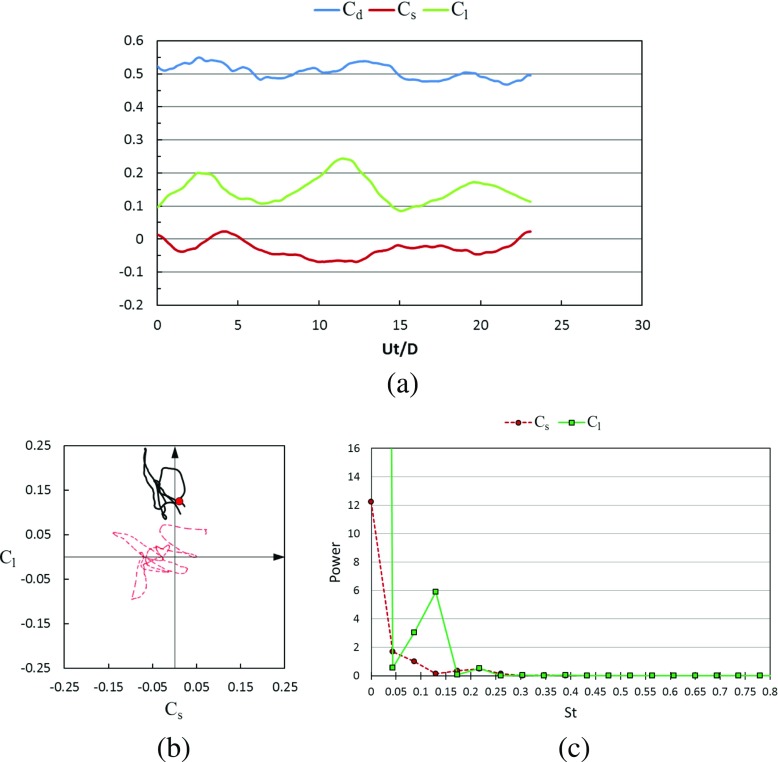
Time series of the drag, side force and lift coefficients of the rotating golf ball at subcritical *R* *e* = 4.3 × 10^4^ after the flow reached a statistically steady state: **a** temporal evolution of the drag coefficient *C*_*d*_, side force coefficient *C*_*s*_ and lift coefficient *C*_*l*_; **b** phase diagram of *C*_*s*_ and *C*_*l*_, the red point represents the resultant lateral force at the non-dimensional moment indicated in Fig. 19; the red dash line represents the corresponding variation of the non-rotating golf ball at the same Reynolds number [8]; **c** power spectrum of the side force coefficient *C*_*s*_ and lift coefficient *C*_*l*_

**Fig. 18:**
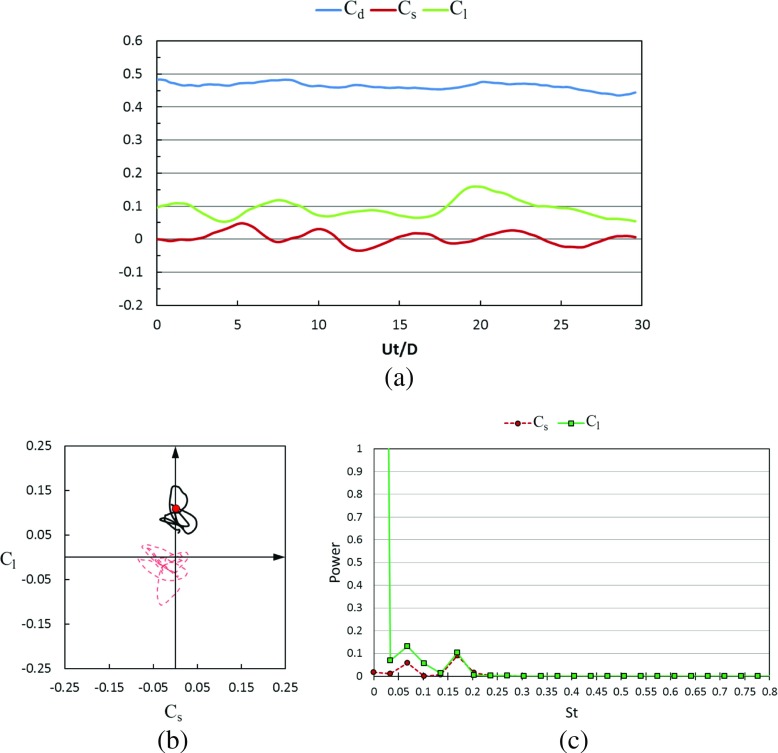
Time series of the drag, side force and lift coefficients of the rotating smooth sphere at subcritical Re = 1.0 × 10^4^ after the flow reached a statistically steady state: **a** temporal evolution of the drag coefficient *C*_*d*_, side force coefficient *C*_*s*_ and lift coefficient *C*_*l*_; **b** phase diagram of *C*_*s*_ and *C*_*l*_, the red point represents the resultant lateral force at the non-dimensional moment indicated in Fig. 20; the red dash line represents the corresponding variation of the non-rotating smooth sphere at the same Reynolds number [8]; **c** power spectrum of the side force coefficient *C*_*s*_ and lift coefficient *C*_*l*_

Figures 17 and 18 show the time histories of the drag coefficient, side force coefficient and lift coefficient in the subcritical Reynolds number regime for the rotating golf ball and the rotating smooth sphere respectively. The phase diagram of the lateral force is also plotted in these figures, which is a good clue to understand the vortex shedding on the surface of the balls and their wake structures. These wake structures strongly affect the separation point of the boundary layer not only in the transient sense but also in the averaged sense. For all the time histories presented in this paper, *U* *t*/*D* = 0 indicates a moment when the flow has reached a statistically steady state [8], where *t* is the physical time. It is revealed by the temporal evolutions that the positive lift forces are continuously acting on both models during the whole time intervals. This makes the variation of the resultant lateral force (the resultant force of side force and lift force) somewhat more regular for the rotating models when compared to the corresponding non-rotating models. As indicated in the phase diagrams, the curves representing the rotating cases (black) stay more concentrated, and exhibit a more dominant oscillation in the lift force direction. For both the rotating and non-rotating cases, the resultant lateral forces acting on the golf ball experience a larger amplitude oscillation when compared to the smooth sphere, as evidenced by the less concentrated curves shown in Fig. 17b and the larger standard deviations of *C*_*s*_ and *C*_*l*_ listed in Table 1. However, the dominant frequency of the lift force oscillation of the rotating golf ball is slightly smaller than that of the rotating smooth sphere, as indicated in Figs. 17c and 18c.

**Fig. 19:**
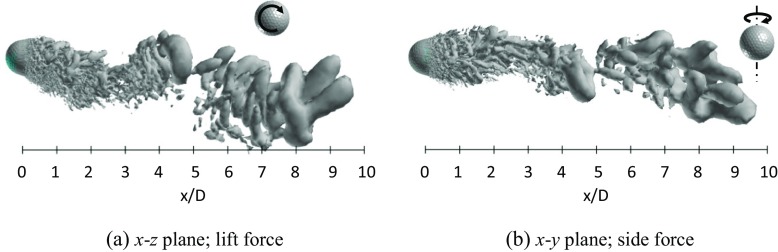
Visualization of the instantaneous vortical structures in the wake area of the rotating golf ball at *R* *e* = 4.3 × 10^4^ using ISO surface of Q (*U* *t*/*D* = 4.9, the resultant lateral force at this moment is indicated in Fig. 17b by the red point)

**Fig. 20:**
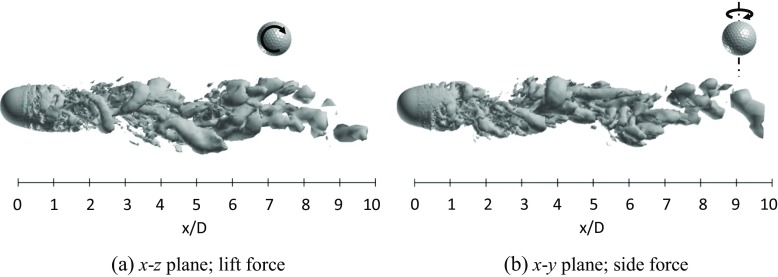
Visualization of the instantaneous vortical structures in the wake area of the rotating smooth sphere at *R* *e* = 1.0 × 10^4^ using ISO surface of Q (*U* *t*/*D* = 21.5, the resultant lateral force at this moment is indicated in Fig. 18b by the red point)

Figures 19 and 20 respectively show the instantaneous vortical structures in the wake areas of the rotating golf ball and the rotating smooth sphere at the subcritical Reynolds numbers. Visualizations were obtained respectively in the lift force plane and the side force plane. As indicated in the figures, the flow structures in the near-wall wake areas of both models are deflected downwards in their lift force planes corresponding to the positive lift forces, especially for the golf ball, which has a larger magnitude of *C*_*l*_. It is interesting to note that the wake flow structure behind the golf ball is also somewhat tilted in the side force plane, whereas the wake flow structure behind the smooth sphere is much less deflected in the same plane. This is probably affected by the specific dimple distribution on the golf ball surface. Similar to the non-rotating cases in the same Reynolds number regime [8], a large-scale wave motion is pronounced in the wake areas of both rotating models, and the hairpin structures become distinct as the flows travel some distance further downstream after full separation. Relating to the corresponding dominant Strouhal numbers, the wavelength of the wake flow structure behind the golf ball spans about 7 ~ 8*D*, as indicated in Fig. 19a, whereas the wavelength of the wake flow structure behind the smooth sphere remains about 6*D*, as indicated in Fig. 20a.

#### Transient lateral forces and wake flow structures in the critical regime

**Fig. 21:**
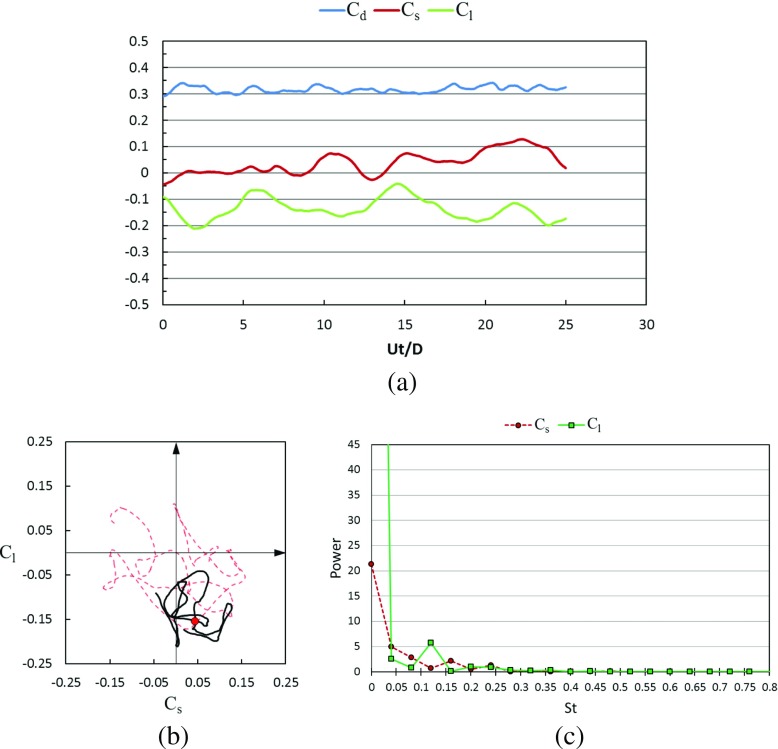
Time series of the drag, side force and lift coefficients of the rotating golf ball at critical *R* *e* = 7.5 × 10^4^ after the flow reached a statistically steady state: **a** temporal evolution of the drag coefficient *C*_*d*_, side force coefficient *C*_*s*_ and lift coefficient *C*_*l*_; **b** phase diagram of *C*_*s*_ and *C*_*l*_, the red point represents the resultant lateral force at the non-dimensional moment indicated in Fig. 23; the red dash line represents the corresponding variation of the non-rotating golf ball at the same Reynolds number [8]; **c** power spectrum of the side force coefficient *C*_*s*_ and lift coefficient *C*_*l*_

**Fig. 22:**
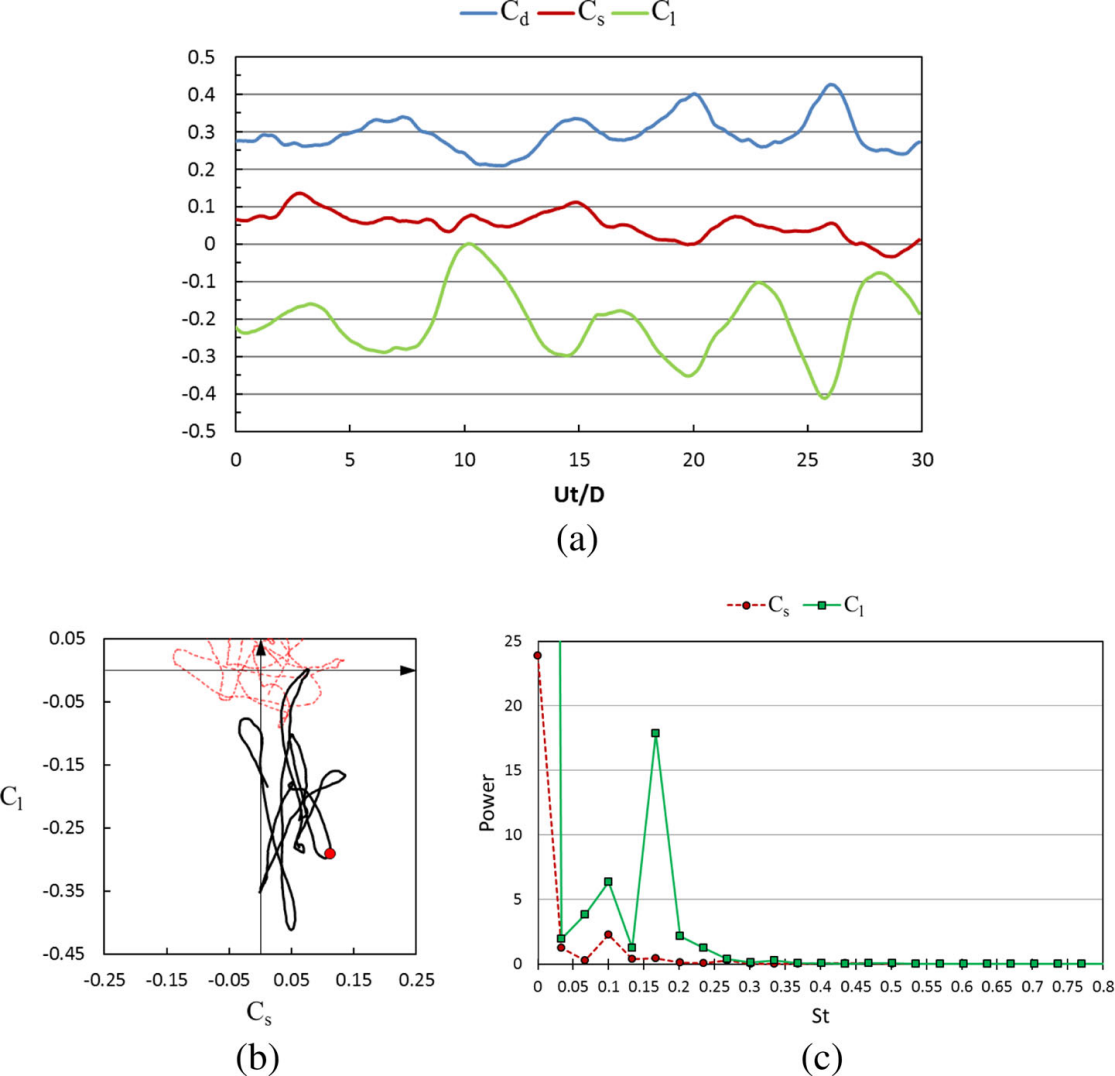
Time series of the drag, side force and lift coefficients of the rotating smooth sphere at critical *R* *e* = 2.0 × 10^5^ after the flow reached a statistically steady state: **a** temporal evolution of the drag coefficient *C*_*d*_, side force coefficient *C*_*s*_ and lift coefficient *C*_*l*_; **b** phase diagram of *C*_*s*_ and *C*_*l*_, the red point represents the resultant lateral force at the non-dimensional moment indicated in Fig. 24; the red dash line represents the corresponding variation of the non-rotating smooth sphere at the same Reynolds number [8]; **c** power spectrum of the side force coefficient *C*_*s*_ and lift coefficient *C*_*l*_

Figures 21 and 22 show the time histories of the drag coefficient, side force coefficient and lift coefficient in the critical Reynolds number regime for the rotating golf ball and the rotating smooth sphere respectively. It is revealed by the temporal evolutions that the negative lift forces are continuously acting on both models during the whole time intervals. This leads to a visible downward-shifting of the mean positions of the resultant lateral force oscillation in the phase diagrams of both models. When compared to the corresponding non-rotating case [8], the resultant lateral force acting on the rotating golf ball experiences a considerably smaller amplitude oscillation, as evidenced by the reduced standard deviations of *C*_*s*_ and *C*_*l*_ listed in Table 1 and the more concentrated curve shown in Fig. 21b. In contrast, the resultant lateral force acting on the rotating smooth sphere experiences a considerably larger amplitude oscillation in the lift force direction when compared to the non-rotating case at the same Reynolds number. This is evidenced by the curves shown in Fig. 22b and the standard deviation of *C*_*l*_ of the rotating smooth sphere which is more than twice as large as that of the non-rotating smooth sphere [8]. The different lateral force variations may further explain the difference in the shifting of drag coefficient between the two models in this Reynolds number regime. As provided in Table 1 and Fig. 4, when compared to the corresponding non-rotating cases, the drag force acting on the rotating golf ball decreases by about 19%, whereas the drag force acting on the rotating smooth sphere increases by about 13%, and has the largest standard deviation among all the rotating cases. It is interesting to note that there are visible side forces acting on both models in this Reynolds number regime even in the time-averaged sense. Similar features were also observed in the experimental measurements of flows past rotating smooth spheres by Kray et al. [15]. This may imply that the negative Magnus effect exhibits somewhat three-dimensional characteristics. Similar to the subcritical cases, the dominant frequency of the lift force acting on the rotating golf ball is smaller than that of the rotating smooth sphere in the critical regime, as indicated in Figs. 21c and 22c.

**Fig. 23:**
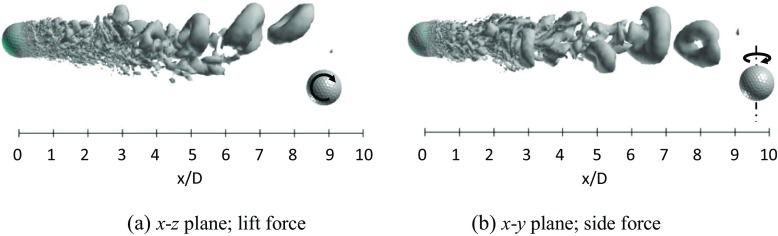
Visualization of the instantaneous vortical structures in the wake area of the rotating golf ball at *R* *e* = 7.5 × 10^4^ using ISO surface of Q (*U* *t*/*D* = 11.7, the resultant lateral force at this moment is indicated in Fig. 21b by the red point)

**Fig. 24:**
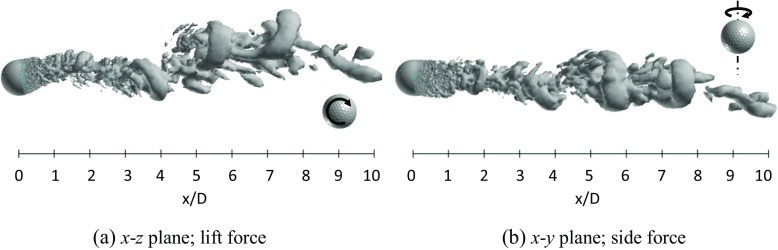
Visualization of the instantaneous vortical structures in the wake area of the rotating smooth sphere at *R* *e* = 2.0 × 10^5^ using ISO surface of Q (*U* *t*/*D* = 14.7, the resultant lateral force at this moment is indicated in Fig. 22b by the red point)

Figures 23 and 24 respectively show the instantaneous vortical structures in the wake areas of the rotating golf ball and the rotating smooth sphere at the critical Reynolds numbers. Visualizations were obtained respectively in the lift force plane and the side force plane. As indicated in the figures, the flow structures in the near-wall wake areas of both models are deflected upwards in their lift force planes corresponding to the negative lift forces, especially for the smooth sphere, which has a larger magnitude of *C*_*l*_. One can also observe that the near-wall wake flow behind both models are somewhat tilted in their side force planes corresponding to the side forces mentioned above. Similar to the non-rotating cases in the critical regime [8], a large-scale wave motion and shed hairpin structures are pronounced in the wake areas of both rotating models, and the waviness behind the smooth sphere is more aggressive due to its larger amplitude oscillation of lift force. Relating to the corresponding dominant Strouhal numbers, the wavelength of the wake flow structure behind the golf ball spans about 8*D*, as indicated in Fig. 23a, whereas the wavelength of the wake flow structure behind the smooth sphere remains about 6*D*, as indicated in Fig. 24a.

#### Transient lateral forces and wake flow structures in the supercritical regime

**Fig. 25:**
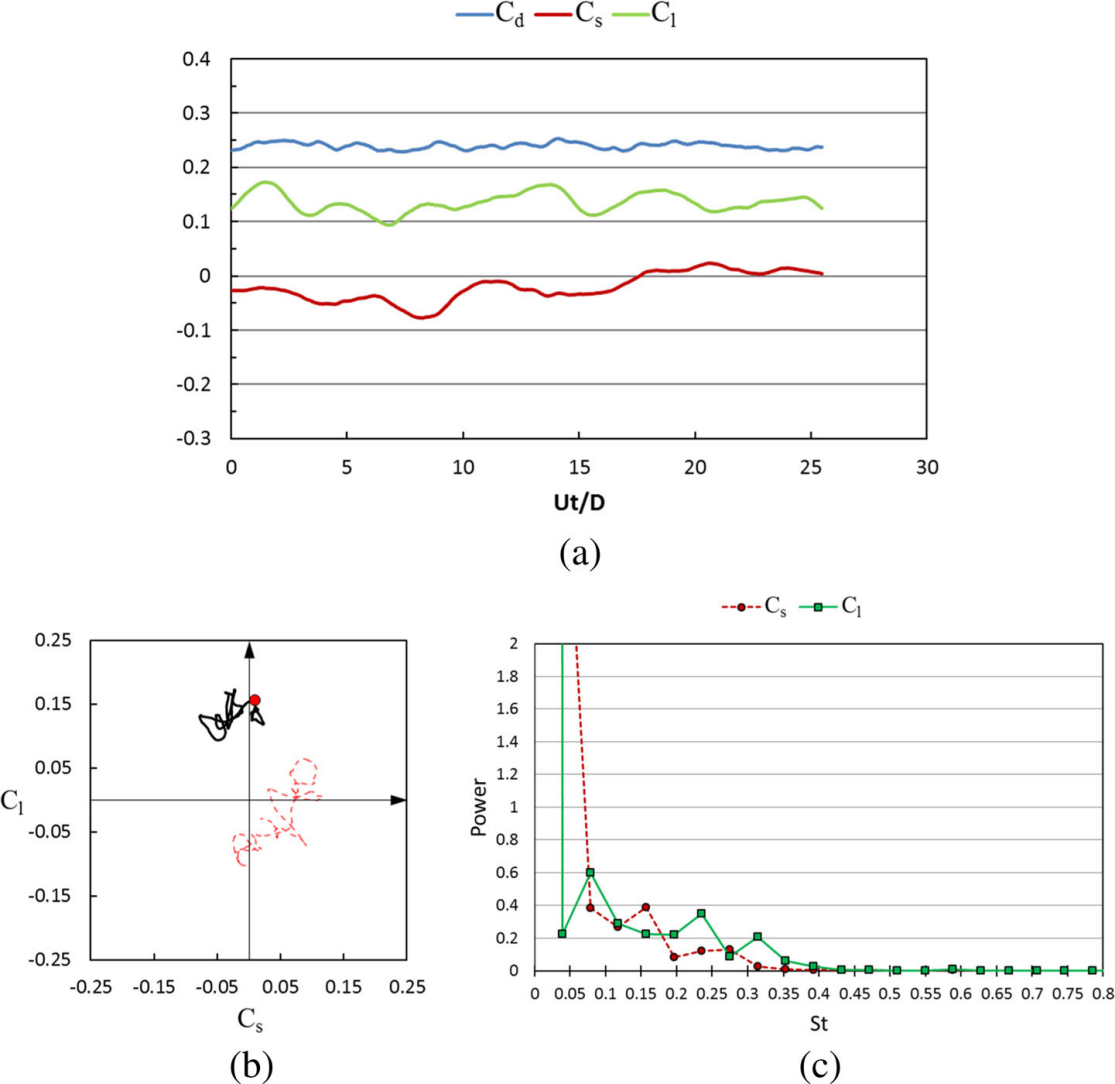
Time series of the drag, side force and lift coefficients of the rotating golf ball at supercritical *R* *e* = 1.1 × 10^5^ after the flow reached a statistically steady state: **a** temporal evolution of the drag coefficient *C*_*d*_, side force coefficient *C*_*s*_ and lift coefficient *C*_*l*_; **b** phase diagram of *C*_*s*_ and *C*_*l*_, the red point represents the resultant lateral force at the non-dimensional moment indicated in Fig. 27; the red dash line represents the corresponding variation of the non-rotating golf ball at the same Reynolds number [8]; **c** power spectrum of the side force coefficient *C*_*s*_ and lift coefficient *C*_*l*_

**Fig. 26:**
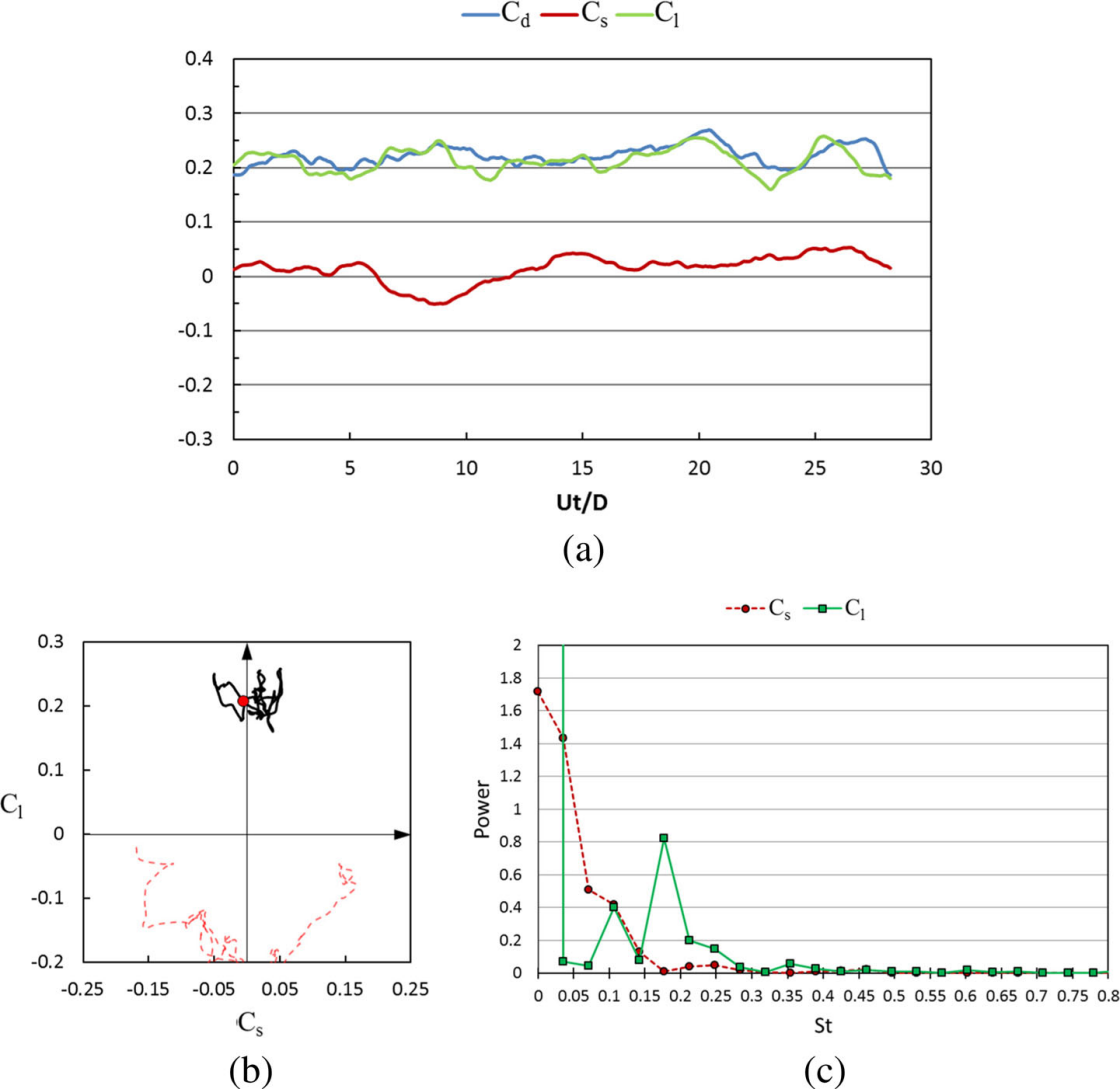
Time series of the drag, side force and lift coefficients of the rotating smooth sphere at supercritical *R* *e* = 1.14 × 10^6^ after the flow reached a statistically steady state: **a** temporal evolution of the drag coefficient *C*_*d*_, side force coefficient *C*_*s*_ and lift coefficient *C*_*l*_; **b** phase diagram of *C*_*s*_ and *C*_*l*_, the red point represents the resultant lateral force at the non-dimensional moment indicated in Fig. 28; the red dash line represents the corresponding variation of the non-rotating smooth sphere at the same Reynolds number [8]; **c** power spectrum of the side force coefficient *C*_*s*_ and lift coefficient *C*_*l*_

Figures 25 and 26 show the time histories of the drag coefficient, side force coefficient and lift coefficient in the supercritical Reynolds number regime for the rotating golf ball and the rotating smooth sphere respectively. It is revealed by the temporal evolutions that the positive lift forces are continuously acting on both models during the whole time intervals. This makes the main areas of the oscillation of the resultant lateral force shift upwards and become more concentrated in the phase diagrams of both models, which is similar to the subcritical cases. When compared to the corresponding non-rotating cases [8], the standard deviations of *C*_*s*_ and *C*_*l*_ of both rotating models reduced significantly. In addition, the rotation trend of the shifting of lateral force direction observed in the corresponding non-rotating cases [8] disappears for both rotating models, as indicated in Figs. 25b and 26b. These features clearly reveal that the rotating motions help to stabilize the lateral forces by diminishing the amplitude of the oscillations and reducing the variance of the directions for both models in the supercritical regime. This is probably because the rotating motions bring about some regularities of the momentum fluctuation in the turbulent boundary layers which consequently leads to a less shifting of the flow separation points. It is also interesting to note that the lift coefficient of the rotating golf ball is about 40% smaller in magnitude and about 20% smaller in standard deviation when compared to the rotating smooth sphere, while the drag coefficients of these two models are similar to each other. These discrepancies further indicate a lift force suppression of the rotating golf ball in this Reynolds number regime, which is analogous to the non-rotating case in the same Reynolds number regime [8]. As revealed in Figs. 25c and 26c, the power spectra of both models remain broadband in this Reynolds number regime. In particular, the dominant frequency of the golf ball that appears at other Reynolds numbers is no longer distinct, which is likely associated to the lift force suppression mentioned above.

**Fig. 27:**
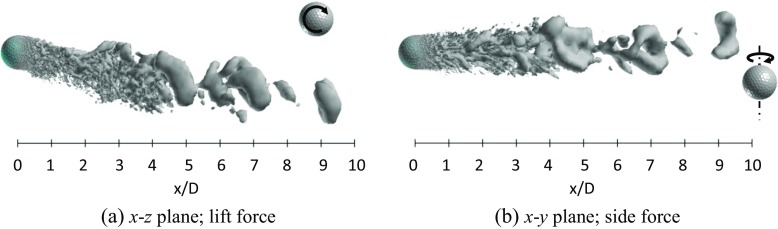
Visualization of the instantaneous vortical structures in the wake area of the rotating golf ball at *R* *e* = 1.1 × 10^5^ using ISO surface of Q (*U* *t*/*D* = 18.8, the resultant lateral force at this moment is indicated in Fig. 25b by the red point)

**Fig. 28:**
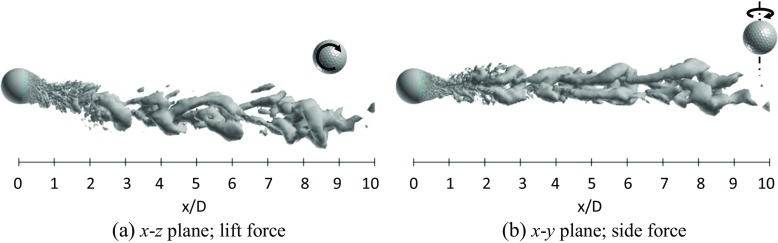
Visualization of the instantaneous vortical structures in the wake area of the rotating smooth sphere at *R* *e* = 1.14 × 10^6^ using ISO surface of Q (*U* *t*/*D* = 6.2, the resultant lateral force at this moment is indicated in Fig. 26b by the red point)

Figures 27 and 28 respectively show the instantaneous vortical structures in the wake areas of the rotating golf ball and the rotating smooth sphere at the supercritical Reynolds numbers. Visualizations were obtained respectively in the lift force plane and the side force plane. As indicated in Figs. 27a and 28a, the wake flow structures of both models are remarkably deflected downwards in their lift force planes corresponding to the positive lift forces. Analogous to the corresponding non-rotating cases [8], a large-scale vertex shedding is pronounced in the wake flows of both rotating models. However, the progressive wave motion exhibited in the subcritical and critical regimes is no longer clearly observed in this Reynolds number regime.

**Fig. 29:**
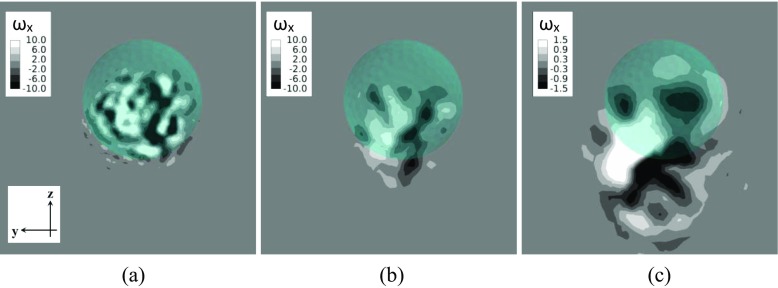
Streamwise vorticity component in the wake flow of the rotating golf ball viewed in the crossflow planes at: **a** *x*/*D* = 1.0; **b** *x*/*D* = 1.5; **c** *x*/*D* = 3.0; *R* *e* = 1.1 × 10^5^(*U* *t*/*D* = 18.8)

**Fig. 30:**
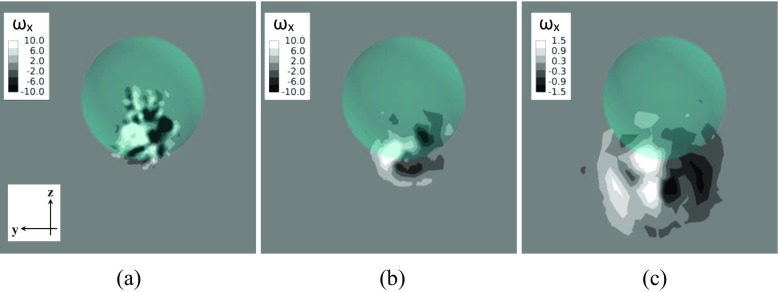
Streamwise vorticity component in the wake flow of the rotating smooth sphere viewed in the crossflow planes at: **a** *x*/*D* = 1.0; **b** *x*/*D* = 1.5; **c** *x*/*D* = 3.0; *R* *e* = 1.14 × 10^6^ (*U* *t*/*D* = 6.2)

Further insights into the differences in the wake structures between the two rotating models in the supercritical regime are provided in Figs. 29 and 30 by the investigation of the streamwise vorticity component *?*_*x*_ (normalized by multiplying *D*/*U*) visualized in the crossflow planes (normal to the freestream direction) respectively located at 1.0*D*, 1.5*D* and 3.0*D* from the center of the golf ball/smooth sphere. As indicated in Fig. 30, a pair of counter-rotating vortices (twin vortices) is clearly shown behind the smooth sphere at each of the three different locations, and it deviates further away from the streamwise axis that goes across the geometry center (*x* axis) along the - *z* direction (due to the positive lift force) as the flow travels downstream. In contrast, the vortices behind the golf ball show more random patterns and a more concentrated distribution around the geometry center at *x*/*D* = 1.0, as shown in Fig. 29a, whereas the twin vortices are detected at locations further downstream (*x*/*D* = 1.5 and *x*/*D* = 3.0), as shown in Fig. 29b and c. It is considered that momentum is generated between the twin vortices along the direction normal to a line connecting the center of each vortex, which consequently gives rise to the lateral forces acting on the models in the opposite direction [8]. When compared to the smooth sphere, the twin vortices appear in the wake flow of the golf ball at a location further downstream, which might be one of the contributions to the weaker lift force acting on the golf ball.

## Conclusions

The flows past a rotating golf ball and a rotating smooth sphere with spin parameter G = 0.1 have been numerically investigated by conducting large-eddy simulations in the subcritical, critical and supercritical Reynolds number regimes. Analogous to the smooth sphere, positive lift forces are generated for the golf ball at the subcritical and supercritical Reynolds numbers, whereas a negative lift force is generated at the critical Reynolds number. When compared to the corresponding non-rotating cases, the drag coefficient of the rotating golf ball increases at the subcritical and supercritical Reynolds numbers while decreases at the critical Reynolds number, whereas the drag coefficient of the rotating smooth sphere increases in all three different Reynolds number regimes.

The mechanism responsible for the negative Magnus effect on the golf ball and the smooth sphere is generally the same: in the critical regime, the boundary layer stays laminar on the top side while it transitions to the turbulent state on the bottom side due to a higher relative Reynolds number. This results in the flow separation on the bottom side occurring at a position further downstream than on the top side, which consequently gives rise to a negative lift force. It should be noted that the laminar-turbulent boundary layer transition on the bottom side is mainly induced by the dimples for the golf ball but mainly caused by the increase in Reynolds number for the smooth sphere.

In the subcritical regime, the resultant lateral forces acting on both rotating models exhibit a more regular oscillation when compared to the corresponding non-rotating cases. The near-wall wake flows are deflected downwards in the lift force planes corresponding to the positive lift forces. A large-scale wave motion and shed hairpin structures are pronounced in the wake flows of both rotating models, as also observed for the corresponding non-rotating models [8].

In the critical regime, when compared to the corresponding non-rotating cases, the resultant lateral force acting on the rotating golf ball experiences a considerably smaller amplitude oscillation, whereas the resultant lateral force acting on the rotating smooth sphere exhibits a considerably larger amplitude oscillation in the lift force direction. This difference in lateral force oscillation might contribute to the different shifting of drag coefficient between these two models. The near-wall wake flows are deflected upwards in the lift force planes corresponding to the negative lift forces. A large-scale wave motion and shed hairpin structures are pronounced in the wake flows of both rotating models, as also observed for the non-rotating models at the same Reynolds numbers [8].

In the supercritical regime, it is discovered that the rotating motions help to stabilize the resultant lateral forces for both models by diminishing the amplitude of the oscillations and reducing the variance of the directions. It is also found that the lift coefficient of the rotating golf ball is about 40% smaller than that of the rotating smooth sphere, whereas the drag coefficients of these two models stay close to each other. The near-wall wake flows are deflected downwards in the lift force planes corresponding to the positive lift forces. A large-scale vertex shedding is pronounced in the wake flows of both rotating models, as also observed for the corresponding non-rotating models [8]. Further investigation on the wake structures reveals the existence of a pair of counter-rotating vortices (twin vortices) behind both models. Particularly, they are detected at a position further downstream for the golf ball, which might be one of the contributions to the weaker lift force acting on the golf ball.

Our final goal of this study is to optimize the dimple geometry and patterns to control the flying path of golf balls. Flight path of various sports from spherical balls to ski jumpers are reviewed by Clanet [16]. Aerodynamic features of other sports balls like cricket ball and baseball, as well as golf balls are summarized by Mehta [17].
